# Patellar fixation graft via suture anchors versus tunnel techniques during isolated MPFL reconstruction for recurrent patellofemoral instability: a systematic review of the literature

**DOI:** 10.1007/s00402-020-03420-8

**Published:** 2020-04-21

**Authors:** Filippo Migliorini, Arne Driessen, Valentin Quack, Hanno Schenker, Markus Tingart, Jörg Eschweiler

**Affiliations:** Department of Orthopaedics, RWTH Aachen University Clinic, Pauwelsstraße 30, 52074 Aachen, Germany

**Keywords:** Patellofemoral instability, MPFL reconstruction, Patellar fixation, Suture anchors, Bone tunnels

## Abstract

**Introduction:**

There is still a lack of evidence concerning the patellar fixation of the medial patellofemoral ligament (MPFL) graft in selected patient with recurrent instability. The purpose of the present study was to investigate and compare clinical outcomes and further complications of isolated MPFL reconstruction via suture anchors versus tunnel techniques for recurrent patellofemoral instability.

**Materials and methods:**

This systematic review of the literature was conducted according to the PRISMA guidelines. In September 2019, the main databases were accessed. All the clinical trials performing isolated MPFL reconstruction in patients with recurrent patellofemoral instability were included in the present study. Only articles fixing the MPFL graft through suture anchors and/ or patellar tunnel techniques were included in the analysis.

**Results:**

Data from 46 papers (1712 patients) were recorded. The mean follow-up was 40 ± 18 months. No differences were found in Kujala, Lysholm and Tegner score, International Knee Documentation Committee, visual analogic scale, range of motion and re-dislocation rate. The suture anchors fixation group detected reduced rate of apprehension test (OR: 0.6; *p* = 0.03), revision surgeries (OR: 0.4; *p* = 0.02) and anterior knee pain (OR: 0.05; *p* < 0.0001) and reduced not-classified complications (OR: 0.18; *p* < 0.0001).

**Conclusion:**

Both the suture anchors and the bone-tunnels are a feasible option for isolated MPFL reconstruction in patients with recurrent patellofemoral instability. Patellar fixation via suture anchors evidenced a reduced rate of anterior knee pain, revision surgeries, apprehension test and an overall reduced complication compared to the bone-tunnel technique.

## Introduction

Patellofemoral instability is a common disorder, especially among young and active patients [1]. Patellofemoral instability is a multifactorial disorder [2]. Several pathoanatomical risk factors that predispose to instability has been described: patella alta, mal-alignment syndromes, axial deformation, patellar dysplasia [3–5]. Most of the patients reported a combination of two or more risk factors that contribute to developing instability [6]. After the first patellar dislocation, tears of medial-patellofemoral ligament (MPFL) occur in approximately in all the patients [7]. Despite the multifactorial etiology, the isolated MPFL reconstruction yields comparable results to the older realignment procedures, with lesser postoperative morbidity and increased patient satisfaction [8]. The role of the MPFL has been extensively investigated in the past decades. However, there is still lack of evidence concerning the patellar fixation of the MPFL graft. Typically, the MPFL reconstruction was performed via bone tunnel techniques. No differences were found in strength between the native MPFL and through tunnel techniques [9]. However, patellar tunnels violate the bone structure, reducing the stability and resistance, leading to an increased risk of secondary fracture [10–13]. In recent times, to avoid tunnelling through the whole length of the patella and related complications, suture anchors techniques have been introduced [14–17]. There are still controversies concerning graft source, positioning, and fixation, and up to date, no consensus has been reached. Hence, the purpose of the present study was to carry out a systematic review of the literature to investigate and compare the role of isolated MPFL reconstruction via suture anchors versus tunnel techniques for recurrent patellofemoral instability.

## Materials and methods

### Search strategy

This systematic review of the literature was conducted according to the Preferred Reporting Items for Systematic Reviews and Meta-Analyses guidelines (PRISMA) [18]. The following criteria were used to guide the literature search:

- P (population): recurrent patellofemoral instability;
- I (intervention): isolated MPFL reconstruction;
- C (comparison): graft fixation techniques;
- O (outcomes): clinical scores, clinical examination, complication.

### Literature search

The literature search was performed by two independent reviewers (FM, JE). In September 2019, the main databases were accessed: PubMed, Medline, Embase, Scopus, and Google Scholar. For the database search, the following keywords and Boolean operators were used in combination: patellofemoral instability, medio-patellofemoral ligament, MPFL, graft, fixation, patellar, femoral, bone, tunnel, suture, anchors, Endobutton, dislocation, re-dislocation, failure, anterior knee pain, Kujala, Tegner, Lysholm, IKDC, range of motion, complications, apprehension test. The full text of the articles of interest was accessed. The bibliographies of the included articles were also screened. Disagreements between the authors were mutually debated and solved.

### Eligibility criteria

All the clinical trials performing isolated MPFL reconstruction in patients with recurrent patellofemoral instability were included in the present study. Only articles describing the graft fixation technique were considered for inclusion. Only articles fixing the MPFL graft through suture anchors and/ or two-patellar tunnel techniques were included in the analyses. According to the author’s language capabilities, only articles in English, Spanish, Italian, German, French were considered for inclusion. According to the Oxford Centre of Evidenced-Based Medicine [19], articles level of evidence I–III were included in the present study. Articles reporting data from acute patellar dislocations were excluded. Articles reporting duration of the follow-up less than 12 months were excluded. Given the quickly evolution of indications and techniques, articles published before the year 2000 were excluded. Articles treating MPFL reconstruction during revision setting or during total knee arthroplasty were excluded. Case reports, expert opinions, editorials, biomechanics, cadaveric and animal studies were excluded. Only articles reporting quantitative Data under the outcomes of interest were included in the present study. Missing data under the outcomes of interest warranted the exclusion from the present study.

### Outcomes of interest

Data extraction was performed by two independent reviewers (FM, JE). The following generalities were extracted: author and year, the number of procedures, mean age of the samples at time of surgery, mean follow-up duration, type of study, graft source, and bundle. The type of surgical techniques and fixations were screened and recorded for each study. Patient outcomes were analysed through the following scores: Kujala Anterior Knee Pain Scale [20], Lysholm Knee Scoring Scale [21], Tegner Activity Scale [22], International Knee Documentation Committee (IKDC) [23], Visual Analogic Scale (VAS), range of motion (ROM). The following complications were recorded: apprehension test, revision surgeries, further re-dislocations, and anterior knee pain. Furthermore, we collected data from other complications (arthrofibrosis, hemarthrosis, subluxation, reduced ROM, quadriceps atrophy, persistent sensation of instability, others).

### Methodological quality assessment

The methodological quality assessment was performed through the PEDro score. This score was performed by two independent authors (FM, JE). The PEDro score has been validated in previous studies [24, 25]. This score evaluated the included studies under 11 dichotomous endpoints. The final value ranked 0–10. Values > 6 are considered satisfactory.

### Statistical analysis

For the statistical analyses, we referred to SPSS software (Version 25, IBM SPSS Statistics). Continuous data were evaluated through the arithmetic mean, standard deviation, and range of intervals. Dichotomous data were evaluated through the odds ratio (OR) statistical method. The interval of confidence was set at 95%. The statistical significance was evaluated through the unpaired *t* test. Values of *p* > 0.05 were considered satisfactory.

## Results

### Search result

**Fig. 1:**
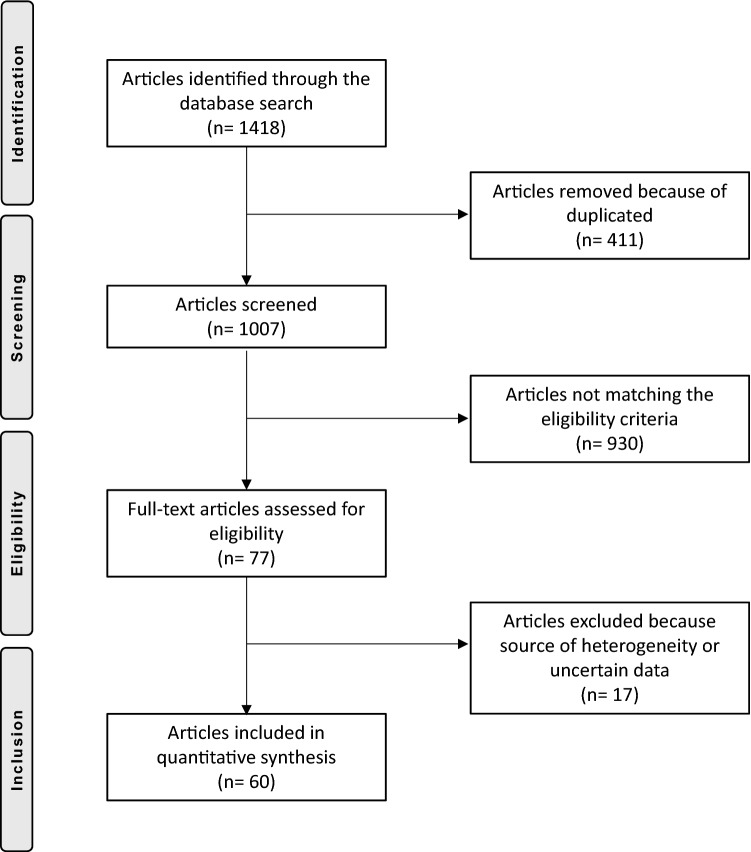
PRISMA flowchart of the literature search

The literature search resulted in 1352 papers. Of them, 398 were rejected because of duplicated. The other 458 studies were excluded because of a poor level of evidence or not performing a clinical study. Further 450 papers were excluded: language incompatibility (51), treating acute dislocations (24), follow-up < 12 months (74); year of publication before 2000 (83), revision setting (31), missing data (77), uncertain results (3), lack of quantitative data under the outcomes of interest (107). Finally, 46 papers were included: 5 randomized clinical trials (RCT), 17 prospective (PCS) and 24 retrospectives (RCS) clinical trials. The literature search is shown in Fig. 1.

### Methodological quality assessment

The PEDro score evidenced some limitations of the present study. First, the lack of randomization and blinding methods among the studies. This will reduce noteworthy the quality of methodological assessment and improve the risk of selection bias. The point of strength was the adequate follow-up of the studies, and the acceptable analyses performed. Concluding, the overall PEDro score resulted in 7.1 points, attesting to the present study a good methodological quality assessment. The PEDro score assigned to each study is shown in Table 1.

**Table 1:** Demographics of the included studies and related PEDro score

Author, year	Type of study	Mean follow-up (months)	PEDro score	Knees (*n*)	Mean age	Patellar fixation	Femoral fixation	Insertion Bundle
Ahmad et al. 2009 [14]	RCS	31	5	20	23	Bone tunnel	Interference screw	Single
Amin et al. 2015 [26]	RCS	24	6	8	22	Bone tunnel	Interference screw	Single
Astur et al. 2015 [27]	RCT	60	8	30	31.06	Bone tunnel	Interference screw	Single
				28	28.32	Suture anchors	Interference screw	Double
Ballal et al. 2018 [28]	PCS	12	7	20	24.4	Suture anchors	Interference screw	NR
Berruto et al. 2014 [29]	PCS	40.6	8	18	NR	Bone tunnel	Interference screw	Double
Carnesecchi et al. 2015 [30]	PCS	25	6	50	23	Suture anchors	Interference screw	Double
Christiansen et al. 2008 [10]	PCS	22	6	32	22	Bone tunnel	Interference screw	Double
Csintalan et al. 2013 [31]	RCS	51	5	56	4.3	Bone tunnel	Interference screw	Double
Feller et al. 2014 [32]	RCS	42	5	26	24	Bone tunnel	Interference screw	Double
Fernandez et al. 2005 [33]	PCS	38	7	30	23	Bone tunnel	Soft tissue	Double
Ellera Gomes et al. 1992 [34]	RCS	39	5	30	28	Bone tunnel	Interference screw	Single
Goncaives et al. 2011 [35]	PCS	26.2	6	22	28.6	Bone tunnel	Interference screw	Double
Han et al. 2011 [36]	RCS	68	6	59	24.3	Bone tunnel	Interference screw	Double
Hiemstra et al. 2017 [37]	RCS	24.4	5	155	25.4	Suture anchors	Suture anchors	Single
Howells et al. 2012 [38]	PCS	16	7	155	26	Bone tunnel	Interference screw	Single
				55	26	Bone tunnel	Interference screw	Single
Kang et al. 2013 [39]	RCT	24	8	40	28.3	Bone tunnel	Interference screw	Double
				42	29.4	Bone tunnel	Interference screw	Double
Kang et al. 2016 [40]	RCT	24	8	23	26.5	Suture anchors	Interference screw	Double
				25	25.6	Suture anchors	Interference screw	Double
Kim et al. 2015 [41]	RCS	19.3	6	9	24.6	Suture anchors	Suture anchors	MIX
Kita et al. 2015 [42]	PCS	39	7	44	25.4	Bone tunnel	Interference screw	Double
Krishna Kumar et al. 2014 [43]	PCS	25	7	30	18	Bone tunnel	Interference screw	Double
Lind et al. 2016 [44]	PCS	39.0	8	24	13	Bone tunnel	Soft tissue	Double
		41.0		179	23	Bone tunnel	Interference screw	Double
Lin et al. 2015 [45]	RCS	35	5	18	NR	Suture anchors	Interference screw	Double
Lippacher et al. 2014 [46]	RCS	25	7	68	18.3	Bone tunnel	Interference screw	Double
Ma et al. 2013 [47]	RCT	40	8	32	28.40	Suture anchors	Interference screw	Double
Matsushita et al. 2014 [48]	RCS	44	6	21	22.10	Suture anchors	Interference screw	Double
		38		18	23.50	Suture anchors	Interference screw	Double
Mikashima et al. 2006 [49]	RCT	41	8	24	21.8	Bone tunnel	Endobutton	Double
Monllau et al. 2015 [50]	RCS	37.6	7	36	25.60	Bone tunnel	Sutured at adductor pedicle	Double
Neri et al. 2014 [51]	RCS	24.3	6	90	22.70	Suture anchors	Interference screw	Double
Niu et al. 2017 [52]	PCS	55.1	7	30	25.00	Bone tunnel	Interference screw	Double
Nomura et al. 2000 [53]	PCS	70.8	7	27	21.00	Bone tunnel	Interference screw	Single
Nomura et al. 2006 [54]	RCS	51	6	12	24.80	Bone tunnel	Suture anchors	single
Nomura et al. 2007 [55]	RCS	143	5	24	22.50	Bone tunnel	Staple	Single
Panni et al. 2011 [56]	RCS	33	5	48	0.25	Bone tunnel	Interference screw or suture anchors	Double
Pinheiro et al. 2018 [57]	RCS	31.2	7	16	27.1	Suture anchors	Interference screw	Single
		34.8		21	26.4	Suture anchors	Interference screw	Single
Raghuveer et al. 2012 [58]	PCS	42	7	15	29.20	Bone tunnel	Interference screw or suture anchors	Single
Ronga et al. 2009 [59]	PCS	37	5	37	28.00	Bone tunnel	Interference screw or suture anchors	Double
Sadigursky et al. 2016 [60]	PCS	12	7	31	29.38	Suture anchors	Interference screw	Double
Schöttle et al. 2005 [15]	RCS	48	6	15	30.10	Suture anchors	Interference screw	Double
Smith et al. 2014 [61]	RCS	12	6	21	23.00	Bone tunnel	Interference screw	Double
Song et al. 2014 [62]	PCS	34.5	7	20	21.00	Suture anchors	Interference screw	Double
Thaunat et al. 2007 [63]	RCS	28	5	23	22.00	Bone tunnel	Suture anchors	Doubled
Toritsuka et al. 2011 [64]	RCS	30	6	20	23.80	Bone tunnel	Endobutton	Doubled
Wang et al. 2010 [65]	RCS	42	7	28	29	Suture anchors	Interference screw	Single
				41	31	Suture anchors	Interference screw	Single
Wang et al. 2013 [66]	RCS	48	8	26	25.00	Suture anchors	Interference screw	Single
				44	25.00	Suture anchors	Interference screw	Double
Wang et al. 2016 [67]	RCS	38	6	26	26.30	Suture anchors	Interference screw	Double
Zhang et al. 2019 [68]	PCS	96	7	60	21	Suture anchors	Interference screw	Double

*RCT* randomized clinical trial, *PCS* prospective cohort study, *RCS* retrospective cohort study

### Demographic data

Data from a total of 1712 patients were recorded. The mean follow-up was 40 ± 18 months. A total of 817 were included in the suture anchors fixation. The mean age of this cohort was 26 ± 3 years. In the double-tunnel technique, a total of 895 knees were analysed, with a mean age of 22 ± 7 years. No differences were found concerning the patient’s age (*p* = 0.08). Demographic data are shown in Table 1.

### Clinical endpoints

No differences were found concerning the Kujala score (87.60 ± 5.2 versus 87.23 ± 6.4, *p* = 0.4), Lysholm score (89.17 ± 4.3 versus 91.51 ± 2.7, *p* = 0.1), Tegner score (5.92 ± 1.2 versus 5.15 ± 0.6, *p* = 0.1), IKDC (72.81 ± 1.6 versus 78.53 ± 5.2, *p* = 0.1), VAS (19.50 ± 2.0 versus 16.88 ± 2.7, *p* = 0.1), ROM (132.14 ± 8.8 versus 132.70 ± 10.9, *p* = 0.5). Noteworthy, analysing the subgroup “double bundle graft”, the only difference was the greater value of the Kujala score in favour of the suture anchors group (89.71 ± 3.5 versus 86.02 ± 6.6, *p* = 0.03). An overview of the clinical results is shown in Table 2.

**Table 2:** Clinical outcome overview

Outcome	Suture anchorss group (*n* = 817)			Double-tunnel group (*n* = 895)			*p*
	Mean	SD	Range	Mean	SD	Range	
Kujala score	87.60	5.2	78–95	87.23	6.4	71–96	0.4
Lyshom score	89.17	4.3	80–95	91.51	2.7	88–95	0.1
Tegner score	5.92	1.2	5–8	5.15	0.6	4–6	0.1
IKDC	72.81	1.6	72–74	78.53	5.2	70–85	0.1
VAS	19.50	2.0	10–25	16.88	2.7	10–20	0.1
Range of motion	132.14	8.8	138–126	132.70	10.9	125–140	0.5

### Complication rate

The suture anchors fixation group detected reduced rate of post-operative apprehension test (OR: 0.5706; 95% CI: 0.3486–0.9338, *p* = 0.03), revision surgeries (OR: 0.4108; 95% CI: 0.1898–0.8890; *p* = 0.02) and anterior knee pain (OR: 0.0522; 95% CI: 0.0126–0.2162; *p* < 0.0001). Re-dislocations were in favour of the suture anchors cohort, but no statistical significance was detected (OR: 0.6086; 95% CI: 0.3215–1.1522; *p* = 0.1). Concerning other not classified complications, the doubled tunnel cohort reported a higher risk (OR: 0.1826; 95% CI: 0.1048–0.3180; *p* < 0.0001). Analysing the subgroup “double bundle graft”, the only differences were found regarding the re-dislocation risk, that were reduced in favour of the suture anchors group (OR: 0.2953; 95% CI: 0.0856–1.0186; *p* = 0.05). An overview of the complication rate is shown in Table 3.

**Table 3:** Complication overview

Complication	Odd ratio (95% confidence interval)	*p*
Apprehension test	0.5706 (0.3486–0.9338)	0.03
Revision surgeries	0.4108 (0.1898–0.8890)	0.02
Anterior knee pain	0.0522 (0.0126–0.2162)	< 0.0001
Re-dislocations	0.6086 (0.3215–1.1522)	0.1
Unspecified complications	0.1826 (0.1048–0.3180)	< 0.0001

## Discussion

The main findings of this systematic review of the literature are that both the suture anchors and the bone-tunnels are a feasible solution for isolated MPFL reconstruction in patients with recurrent patellofemoral instability. MPFL reconstruction through suture anchors reported a statistically significant reduced rate of postoperative anterior knee pain, along with an overall reduced complication compared to the bone-tunnels technique. Moreover, the subgroup suture anchors via double-bundle graft detected a statistically significant reduction of the re-dislocations rate and a minimal improvement of the Kujala score compared to the double tunnel technique. No differences were found in terms of ROM and clinical scores.

Concerning the clinical scores, only the subgroup suture anchors fixation through double-bundle MPFL graft evidenced a significantly greater value of the Kujala score over the bone tunnel techniques. Contextualizing, these data found no clinical relevance, since the small difference of 3.69%. All the other scores of interest detected similarity among the two techniques. Concerning complications, the suture anchors fixation group detected an overall reduction of the complications. The outcomes apprehension test and revision surgeries detected a significant halved risk in the suture anchors group respect to the bone tunnel cohort. Noteworthy, the risk of developing anterior knee pain was strongly reduced in the suture anchors group. The re-dislocation rate of the suture anchors versus bone tunnel fixation was similar. Interestingly, the analysis of the subgroup anchor fixation via double-bundle showed a significantly reduced re-dislocations risk of about one-third compared to the bone tunnel techniques.

In the literature, there is a lack of clinical studies comparing directly suture anchors fixations and bone tunnel techniques. Kang et al. [69] performed a systematic review of the literature including 21 studies, consisting of 691 patients undergoing 36-month follow-up. They found no differences among the two techniques concerning Kujala, apprehension test, dislocations, and complications. Several studies analysed the biomechanics of the various patellar insertion techniques. From a biomechanical point of view, the suture anchors evidenced lower stiffness than the tunnel techniques (21 N/mm versus 28 N/mm), while no differences were found in the ultimate load (299 N versus 304 N, respectively) [70]. The biomechanical study of Lenschow et al. [71] evaluated the maximum load to failure and elongation. The suture anchors showed a higher maximum load to failure than the bone tunnel techniques (401 Nm versus 354 Nm), better elongation after 1000 cycles (3.7 mm versus 1.9 mm) [71]. He et al. [72] compared the native MPFL reconstruction versus double and single bundle. They found similarity between single bundle and native MPFL in terms of tensile strength (146 N versus 159 N) and elongation (8.39 mm versus 7.64 mm) [72]. In their study, they found a considerably higher tensile strength and elongation in double-bundle suture anchors group (314 N and 12 N, respectively) [72]. In the cadaveric study of Mountney et al. [9], the load to failure was tested of the native MPFL versus several different MPFL repair and reconstruction in ten knees. They found that suture anchors showed lower failure strength than the native MPFL [9].

The higher complexity of this topic, along with the controversial and reduced knowledge concerning the patellofemoral disorders considerably pose important limitation to draw solid conclusions. Even if the overall methodological assessment via the PEDro score resulted acceptably, an important limitation of the present study was the overall low quality of the included studies. Only one-tenth of the studies provided a randomization allocation, no one took advantage of a blinding method. This increases considerably the risk of selection bias, therefore, data from this study must interpret with caution. The following study analysed outcomes and complications with regard to the patellar fixation exclusively. Type of graft, femoral insertion and tensioning were not considered. This represents another important limitation of the present work. The good baseline comparability and the comprehensive nature of the literature search, along with the strict eligibility criteria represented the most important point of strength of this study.

## Conclusion

Both the suture anchors and the bone-tunnels are a feasible solution for isolated MPFL reconstruction in patients with recurrent patellofemoral instability. MPFL reconstruction through suture anchors reported a statistically significant reduced rate of postoperative anterior knee pain, apprehension test and revisions, along an overall reduced complication compared to the bone-tunnels technique. Moreover, the subgroup suture anchors via double-bundle graft detected a statistically significant reduction of the re-dislocations rate and a minimal improvement of the Kujala score compared to the double tunnel technique. No differences were found in terms of ROM and clinical scores.
